# Effect of a community-based approach of iron and folic acid supplementation on compliance by pregnant women in Kiambu County, Kenya: A quasi-experimental study

**DOI:** 10.1371/journal.pone.0227351

**Published:** 2020-01-10

**Authors:** Mary Wanjira Kamau, Samuel Thuo Kimani, Waithira Mirie, Isaac Kamau Mugoya

**Affiliations:** 1 School of Nursing Sciences, University of Nairobi, Nairobi, Kenya; 2 John Snow Inc., Nairobi, Kenya; University of Dhaka, BANGLADESH

## Abstract

**Introduction:**

Iron and Folic Acid Supplementation (IFAS) is an essential and affordable intervention strategy for prevention of anaemia during pregnancy. The supplements are currently provided for free to pregnant women in Kenya during antenatal care (ANC), but compliance remains low over the years. There is need for diversification of IFAS programme implementation by exploring other distribution channels to complement existing antenatal distribution and ensure consistent access to IFAS supplements.

**Objectives:**

To determine the effect of a community-based approach of IFAS distribution on compliance and assess side-effects experienced and their mitigation by pregnant women in Kiambu County.

**Methodology:**

A pretest-posttest quasi-experimental study design was used, consisting of an intervention and a control group, among 340 pregnant women 15–49 years, in five health facilities in Lari Sub-County in Kiambu County, between June 2016 and March 2017. Community health volunteers provided IFAS supplements, counselling and weekly follow-up to pregnant women in the intervention group while the control group followed standard practice from health facilities. Baseline and endline data were collected during antenatal care and compared. Quantitative data was analyzed using STATA version 14. Analysis of effect of intervention was done using Difference-In-Difference regression approach.

**Results:**

Levels of compliance increased by 8% in intervention group and 6% in control group. There was increased awareness of IFAS side-effects across groups. The intervention group reported experiencing less side-effects and were better able to manage them compared to the control group.

**Conclusion:**

Implementation a community-based approach improved maternal compliance with IFAS, awareness of IFAS side effects and their management, with better improvement being recorded in the intervention group. Hence, there is need to integrate community-based approach with antenatal distribution of IFAS to improve supplementation.

## 1. Introduction

Iron and folic acid are essential micronutrients whose deficiency causes anaemia [1] especially during pregnancy when the demand for these nutrients is higher than usual. With the global prevalence of between 41.8% and 43.8% [2], anaemia during pregnancy is a major contributor to the global burden of disease. The prevalence of anaemia is highest in Africa at 61.3% followed by 52.5% in South East Asia [3]. In developing countries, it is estimated that every second pregnant woman is anaemic [4]. In Kenya, anaemia in pregnancy remains a public health problem with 55.1% of the pregnant women being anaemic [5], and iron deficiency accounting for approximately 60% [6].

If not addressed, anaemia, can eventually cause maternal, foetal and infant death. Globally, 1 out of 5 maternal deaths are associated with anaemia [7]. In Kenya, anaemia in pregnancy is associated with estimated 1 in 5 perinatal deaths and 1 in 10 maternal deaths respectively [8]. A great proportion of these deaths can be averted by strengthening Iron and Folic Acid Supplementation (IFAS) and increasing its compliance by pregnant women. Iron and folic acid supplementation has been shown to be beneficial by reducing maternal and infant morbidity (including postpartum haemorrhage and poor birth outcomes, such as preterm births and low birth weights) and mortality associated with anaemia [1, 9].

Iron and folic acid supplementation increases haemoglobin levels thereby reducing anaemia rates and improving birth outcomes [1, 10]. Research shows that it reduces risk of maternal anaemia and iron deficiency at term by 70% and 57% respectively [11]. In addition, supplementation with Iron and Folic Acid (IFA) tablets can increase the mean blood haemoglobin level by 10·2 g/l in pregnant women and by 8·6 g/l in non-pregnant, consequently eliminating about 50% of anaemia in women [12, 13] as well as anaemia risk among infants [14]. Furthermore, intake of 90–150 IFA tablets can decrease neonatal mortality by 34%-45% [15–17]. Maternal and child mortality are two most important indicators of health, which if improved, would go a long way in achieving Universal Health Coverage (UHC) against anaemia, in line with the mission of UHC 2030 to accelerate both equitable and sustainable progress towards UHC globally. Consequently, this will contribute to achievement of United Nations Sustainable Development Goal 3: Good health and well-being for all.

The current mode of distribution of IFA tablets through health facilities only has not been able to attain the required coverage. There is therefore need to diversify IFAS distribution channels. Following the World Health Organization (WHO) recommendations, Kenya adopted oral iron and folic acid supplementation programme in 2010, as a high impact nutrition intervention to specifically control anaemia in pregnancy [18]. Kenya’s efforts to improve uptake of IFAS have included adoption of a combined iron and folic acid tablet in 2012 and provision of the supplements free of charge to pregnant women at all public health facilities. However, compliance with IFAS has remained low over the years and prevalence of anaemia in pregnancy remains persistently high (55.1%) [5]. The 2014 Kenya Demographic Health Survey indicated that less than 8% pregnant women took IFA supplements for 90 or more days and over 30% did not take them at all [19]. Community based distribution of IFA tablets coupled with closer follow-up through home visits, if adopted, can play a role in improving compliance and eventually reducing the burden of anaemia in pregnancy.

Studies have shown that side effects to daily IFA tablets are a major reason for low compliance and a barrier to full realization of benefits of supplementation [3, 20–23]. The most mentioned sides effects include gastrointestinal (such as epigastric pain, nausea, vomiting, diarrhea, constipation or gastritis) and dark stools [5, 18, 24]. However, most studies done do indicate the IFAS side-effects that pregnant women experience but not how they mitigate or handle them. Clients concerns are better addressed during issuance of the tablets. However, many health care providers do not even inform clients that they may experience side effects and clients become non-compliant with any slight side effect experienced [5]. A previous study indicated that the severity and frequency of occurrence of side effects increases with the amount/dose of iron administered [25], although this variation was not observed when the dose was less than 100mg. Consequently, the combined IFA tablet has less amount of iron per tablet (60mg) compared with iron only tablet (200mg). This when combined with folic acid into one tablet reduces both side effects experienced by pregnant women and pill burden. This was expected to increase compliance by pregnant women, but as indicated earlier, the compliance remains low nationally.

Previous studies recommended adoption of community-based delivery of IFAS [26] and its integration into the formal health-care system [25] because of its contribution in improving compliance. Experiences in Nicaragua, Gambia, Indonesia and Thailand, have proved that community based IFAS distribution can help achieve higher rates of compliance by reaching more women than antenatal (ANC) distribution alone [27]. Further evidence from Ghana and Malawi indicate that community involvement in anaemia control interventions is critical to their success [28, 29]. Community based interventions involve use of volunteers/community agents, as point of contact/intermediary between intervention/health service and clients at community level. Indeed, since the beginning of the 21st century, many health programs started utilizing Community Health Workers (CHWs) because of their unique capacity to connect community members with appropriate health care services. The CHWs serve as "bridges" between the health care services and community members. The CHWs can develop partnerships with formal health care delivery systems and work with health care providers to support community participation in health activities for the success of health programmmes.

In developing countries, two factors that have been identified to substantially contribute to low compliance with IFAS during pregnancy are poor awareness about IFAS and anaemia as well as insufficient health delivery systems [3]. In Kenya, IFAS is currently provided during antenatal care clinic visits only. Adequacy of antenatal care is a major determinant of IFAS utilization during pregnancy [30]. Studies have shown that low access and poor utilization of antenatal care (ANC) services is highly associated with low utilization of IFAS [31, 32]. Most pregnant women in Kenya start ANC services late [8] and therefore do not benefit optimally from the IFAS. This points to the need to diversify delivery strategies beyond ANC attendance in order to reach more women. The best health outcomes have been realized when the supplementation uses community-based distribution systems [33]. Use of CHWs for IFAS distribution is one such option.

Research has shown that CHWs are able to identify pregnant women early in pregnancy and can therefore provide supplements to these women early in pregnancy when they are most beneficial [7, 9]. Community health workers are in constant touch with the women and can therefore provide regular and consistent follow up and improve compliance. This approach has not been previously used in Kenya. There remains a gap in integration of community based with formal IFAS distribution strategies in Kenya, which formed the basis for this study. In an effort to bridge this gap and address the insufficient IFAS delivery channel, this study aimed at introducing a community based IFAS distribution strategy to complement the existing health facility based IFAS distribution in order to increase its utilization by pregnant women in the community. This study used CHWs, otherwise referred to as Community Health Volunteers (CHVs) in Kenya to distribute IFAS tablets to the pregnant women in their homes and to counsel them. Therefore, the objectives of this study were to (1) determine the effect of a community based IFAS distribution on compliance (2) identify IFAS side-effects experienced by pregnant women and (3) assess mitigation measures of IFAS side-effects by pregnant women, before and after community-based distribution of IFAS.

## 2. Materials and methods

### 2.1 Study site

This study was conducted between June 2016 and March 2017, in Kenya, Kiambu County, Lari Sub-County, in five of its major public health facilities (Lari, Githirioini, Kagwe, Kagaa and Kinale).

### 2.2 Study design and structure

**Fig 1:**
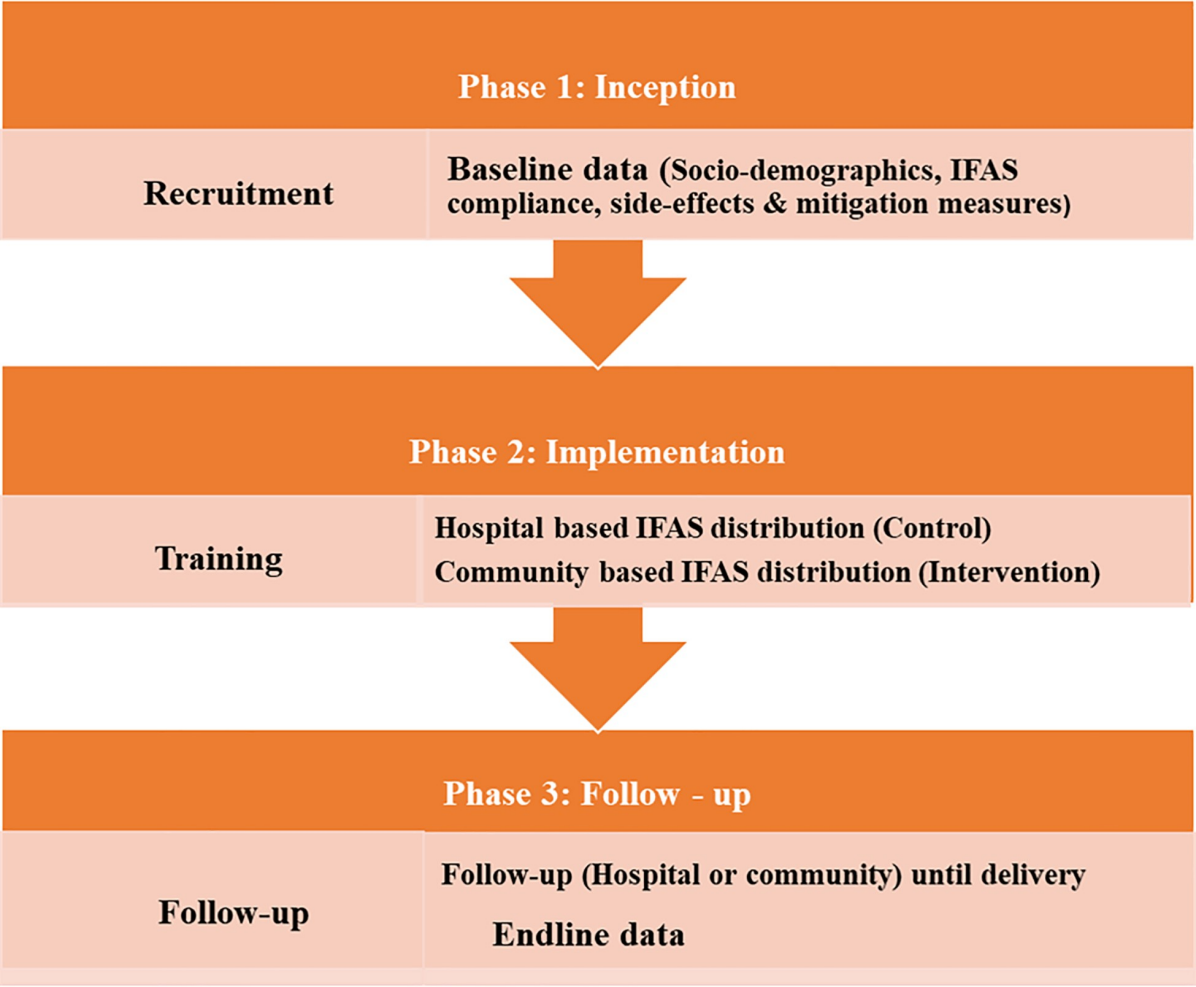
Study phases. This figure shows the three stages that were followed in the implementation of this study.

This was a pretest-posttest quasi-experimental study design with a control group. The study involved three phases as shown in Fig 1, namely inception, implementation and follow-up phases. Inception was the first phase and involved identification and recruitment of study respondents as well as baseline data collection including: socio-demographic characteristics, compliance with IFAS, side-effects experienced with IFAS and their mitigation measures by pregnant women. Implementation was the second phase and involved training of Health Care Providers (HCPs), mostly nurses and Community Health Volunteers (CHVs) on IFAS programme then distribution of IFAS supplements with counselling information. The control group followed standard routine practice of receiving IFAS tablets from HCPs during antenatal care clinics. The intervention group received IFAS tablets from CHVs who distributed the IFAS tablets to pregnant women in their homes. Follow-up was the third phase and involved following the pregnant women up to delivery of their babies as well as collection of endline data. The control group were followed up by HCPs (specifically nurses) during routine antenatal care in health facilities. The intervention group were followed up by CHVs on a weekly basis in their homes. During the weekly home visits, CHVs provided each pregnant woman with the entire week’s supply of IFAS tablets, evaluated previous week’s intake and counselled her on various IFAS topics including common side effects such as black stools, stomach upset, constipation and diarrhea, and their mitigation measures. In addition, the CHVs encouraged pregnant women to attend antenatal care clinics to receive the other antenatal care services. Using a similar questionnaire like that used to collect baseline data, endline data was then collected before delivery, from 36^th^ week of gestation, in both control and intervention groups.

### 2.3 Sampling and study population

Some of the details of methods adopted for recruitment of the pregnant women involved in this study as well as the ethical considerations have been published elsewhere in other articles [34, 35].

From Kiambu County, two stage sampling method was used to select one Sub-County (Lari) and five of its major public health facilities (Lari, Githirioini, Kagwe, Kagaa and Kinale). The sampling frame consisted of all Sub-Counties in Kiambu County. Lari Sub-County was selected on the basis of having existing functional (active) community units, meaning its community health volunteers were actively involved in provision of community health services to community members. The five health facilities were selected on the basis of existing functional (active) community units attached to them and high client population turnover, due to the low turnover of ANC clients.

The study sample size was calculated using the following formula for a binary outcome [36]:

$$n=(\frac{r+1}{r})\frac{(\bar{p})(1-\bar{p}){\left(Z_{\beta }+Z_{\alpha /2}\right)}^{2}}{{\left(D\right)}^{2}}$$

D is the expected effect in IFAS compliance of 20% (control 25% to 45% intervention)

A consideration of 30% loss to follow-up was added to this sample, making a total sample size of 170. The final sample size per study group was 170 and in both groups was therefore **340**.

The study population consisted of all pregnant women who attended antenatal care clinic in the five health facilities. The inclusion criteria was: age 18–49 years, below 33 weeks in their pregnancy gestation, not suffering from any chronic illness and who provided informed consent to participate in the study. Consecutive sampling method, which is considered the best type of non-probability sampling with best representation of entire population, was used to include all accessible pregnant women as part of the sample. All pregnant women who met the inclusion criteria were informed about the study and those who provided both verbal and written informed consent to participate in the study were recruited. Those residing in a community that had a functional community unit with active community health volunteers who consented to have IFAS distributed to them in their homes formed the intervention group. Those who consented to participate and were residing in a community that did not have a functional community unit, formed the control group, who received their IFAS from fixed health facilities during antenatal care clinics, until the required sample size of 170 was reached.

### 2.4 Data collection tools and process

A semi-structured interviewer-administered questionnaire consisting of 23 closed ended questions including; 11 on socio-demographic data, and 12 on IFAS utilization at health facility, was developed, pre-tested and used for data collection in this study. To address any potential bias in data collection, four research assistants were trained on research ethics, protocols and quality data collection at Kiambu level 5 hospital where the research questionnaires were pretested.

To ensure reliability of the questionnaire, a test re-test method was adopted in pre-testing, whereby a repeat pre-test was conducted after two weeks, and Cohen’s kappa statistic was used to measure the level of agreement of the results from the two pre-tests. The questions which were re-tested included: on socio-demographic data: age, education level, occupation, income, gestation, parity and gravidity; on IFAS utilization: frequency of taking IFAS, duration of taking IFAS, timing when IFAS was taken, number of tablets taken in the past 7 days, IFAS side effects experienced and how the pregnant women mitigated the side effects. All the questions repeated had a kappa value of above 0.7 after comparison thus the questionnaire was considered reliable, hence all the questions were retained. To ensure validity of the questionnaire, it was shared and discussed with experts from the Ministry of Health, division of nutrition, and the study supervisors. The feedback obtained from these experts and pre-testing results was used to refine the tool and improve its quality to ensure the questions were able to test what was intended.

The trained research assistants then administered questionnaires to all pregnant women who met the inclusion criteria and consented to participate in the study.

### 2.5 Determination of respondents’ compliance levels

The compliance with IFAS was assessed based on the reported number of IFAS tablets taken in the preceding one week (seven days) before the interview. The IFAS compliance status was defined as the number of IFAS tablets taken in the preceding seven (7) days. Pregnant women who took at least 70% of the expected dose of the IFAS tablets in the week preceding the interview, an equivalent of five IFAS tablets per week, were considered compliant with IFAS [37, 38]. Conversely, the respondents who took less than 30% of the expected IFAS dose, an equivalent of less than five IFAS tablets, were considered non-compliant. Women who had attended the first ANC visit in the current pregnancy and reported not to have received IFAS tablets before, were excluded in the assessment of compliance since pregnant women routinely receive IFAS during ANC visits.

### 2.6 Data management and analysis

In order to examine effectiveness of the intervention, baseline and endline surveys were conducted in both study groups, using a similar questionnaire. To ensure adherence to optimal data quality standards, the researcher closely supervised the research assistants.

Quantitative data at both baseline and endline was coded after collection then entered into the computer, cleaned and validated using Statistical Package for Social Sciences (SPSS) statistical software version 22. Data entry was done during the study data collection process to minimize errors. The entered data was then exported to STATA version 14 for analysis. To ensure confidentiality, the computer access was restricted by password protection. Each questionnaire had a unique identifier to allow validation. Data cleaning and validation was done prior to analysis.

Descriptive statistics, including univariate analysis: simple proportions, n (%), for categorical variables and mean with standard deviation for continuous variables, were reported at baseline and endline. Characteristics of respondents were also described in both intervention and control groups. To ensure the change caused by the intervention was not by chance, similarity of baseline characteristics in both groups was ascertained. Homogeneity of study groups at baseline was determined by comparing socio-demographic characteristics of both groups. Side-effects experiences and the measures applied by pregnant women to mitigate them were recorded. Bivariate analysis, using the chi-square test, was done for comparison between groups and multivariate analysis was used to control for confounders.

The analysis of effect of the intervention was done using a Difference-In-Difference (DID) regression model to compare outcomes between intervention and control groups before (baseline) and after (endline) the intervention. The changes in the dependent variables in the intervention group (from baseline to endline) were compared to changes in the control group (from baseline to endline) as shown in Table 1 below [39]. The intervention effect was measured by odds ratio and 95% confidence level of the interaction term between study groups (intervention and control) and period of survey (baseline and endline) in the regression model. A p-value of 0.05 was considered statistically significant. Since the same respondents who participated in the baseline are the same who participated in the end term evaluation, the analysis adopted a paired analysis with repeated measures instead of treating the respondents in baseline and endline as independent groups.

**Table 1:** Intervention effect formula.

	Baseline		Endline
Intervention group	Level of phenomenon before intervention (X)	Intervention introduced	Level of phenomenon after intervention (Y)
Control group	Level of phenomenon without intervention (A)		Level of phenomenon without intervention (Z)
	**Intervention Effect = (Y-X)–(Z-A)**		

Source: Kothari and Garg, 2014 pg 41

### 2.7 Ethical considerations

Scientific and ethical approval was sought and obtained from Kenyatta National hospital/University of Nairobi Ethics and Research Committee (KNH-ERC/A/90 protocol number–P706/11/2015). Research permit was sought and obtained from the National Commission for Science, Technology and Innovation (NACOSTI/P/18/81499/22319). Authority to conduct the study was obtained from Kiambu County, Lari Sub-county authorities and all health facilities involved.

Respondents were fully protected from any form of harm. Participation in the study was purely voluntary. The purpose of study was clearly explained to respondents who were required to provide informed verbal and written consent. Emphasis on confidentiality and privacy were made clear at the time of consenting to participate and upheld throughout the study. No name appeared on the questionnaires so no participant identification with information could occur. Respondents were at liberty to discontinue from the study at any time without facing any adverse consequences. Information was kept confidential by restricted access and coding of questionnaires.

## 3. Results

### 3.1 Socio-demographic characteristics of study respondents

A total of 340 pregnant women participated in the study during the baseline, of these 189 (56%) participated during the endline. Table 2 shows the socio-demographic characteristics of study respondents at baseline according to study group. Most (n = 212, 62%) respondents were 20–29 years of age, with mean age of 25.6 (SD ± 5.6), had a secondary level of education (n = 180, 53%) and unemployed (n = 167, 49%). Majority of them were married (n = 288, 85%) and earned less than USD. 100 per month (n = 305, 93%). Whereas only 6% (n = 20) had attained tertiary level of education, only 3% (n = 10) were formally employed. In terms of gravidity, most (n = 223, 68%) of the women were multigravida. The study groups were homogeneous at baseline (Table 2) since there was no statistical difference (p>0.05) in the baseline characteristics of respondents between the two comparison groups.

**Table 2:** Socio-demographic profile of the study respondents at baseline by group.

Variable	Total (Col %) N = 340	Hospital (Row %) N = 218	Community (Row %) N = 122	Chi-square p-value
**Age of pregnant woman in years**				
Less than 20 years	43 (12.6)	27 (62.8)	16 (37.2)	0.899
20–29 years	212 (62.4)	138 (65.1)	74 (34.9)	
30 years and above	85 (25)	53 (62.4)	32 (37.6)	
Mean age (std)	25.6 (5.6)	25.6 (5.9)	25.7 (5.7)	
**Marital status**				
Married	288 (84.7)	183 (63.5)	105 (36.5)	0.32
Single	51 (15)	35 (68.6)	16 (31.4)	
**Education level**				
Primary	137 (40.7)	89 (65)	48 (35)	0.467
Secondary	180 (53.4)	111 (61.7)	69 (38.3)	
Tertiary	20 (5.9)	15 (75)	5 (25)	
**Occupation of pregnant woman**				
Unemployed	167 (49.1)	108 (64.7)	59 (35.3)	0.978
Casual employment	77 (22.6)	49 (63.6)	28 (36.4)	
Self-employed/Employed	96 (28.2)	61 (63.5)	35 (36.5)	
**Average income per month in USD**				
Less than100	305 (93)	194 (63.6)	111 (36.4)	0.274
100 and above	23 (7)	12 (52.2)	11 (47.8)	
**Parity**				
0	111 (33)	74 (66.7)	37 (33.3)	0.106
1	92 (27.4)	53 (57.6)	39 (42.4)	
2	80 (23.8)	50 (62.5)	30 (37.5)	
3 and above	53 (15.8)	41 (77.4)	12 (22.6)	
**Gravidity**				
Primigravida	107 (32.4)	74 (69.2)	33 (30.8)	0.171
Multigravida	223 (67.6)	137 (61.4)	86 (38.6)	
**Religion of pregnant woman**				
Protestant Christian	283 (83.5)	179 (63.3)	104 (36.7)	0.512
Catholic Christian	56 (16.5)	38 (67.9)	18 (32.1)	

### 3.2 Effect of community-based approach on maternal compliance with IFAS

#### 3.2.1 Compliance with IFAS among pregnant women

**Fig 2:**
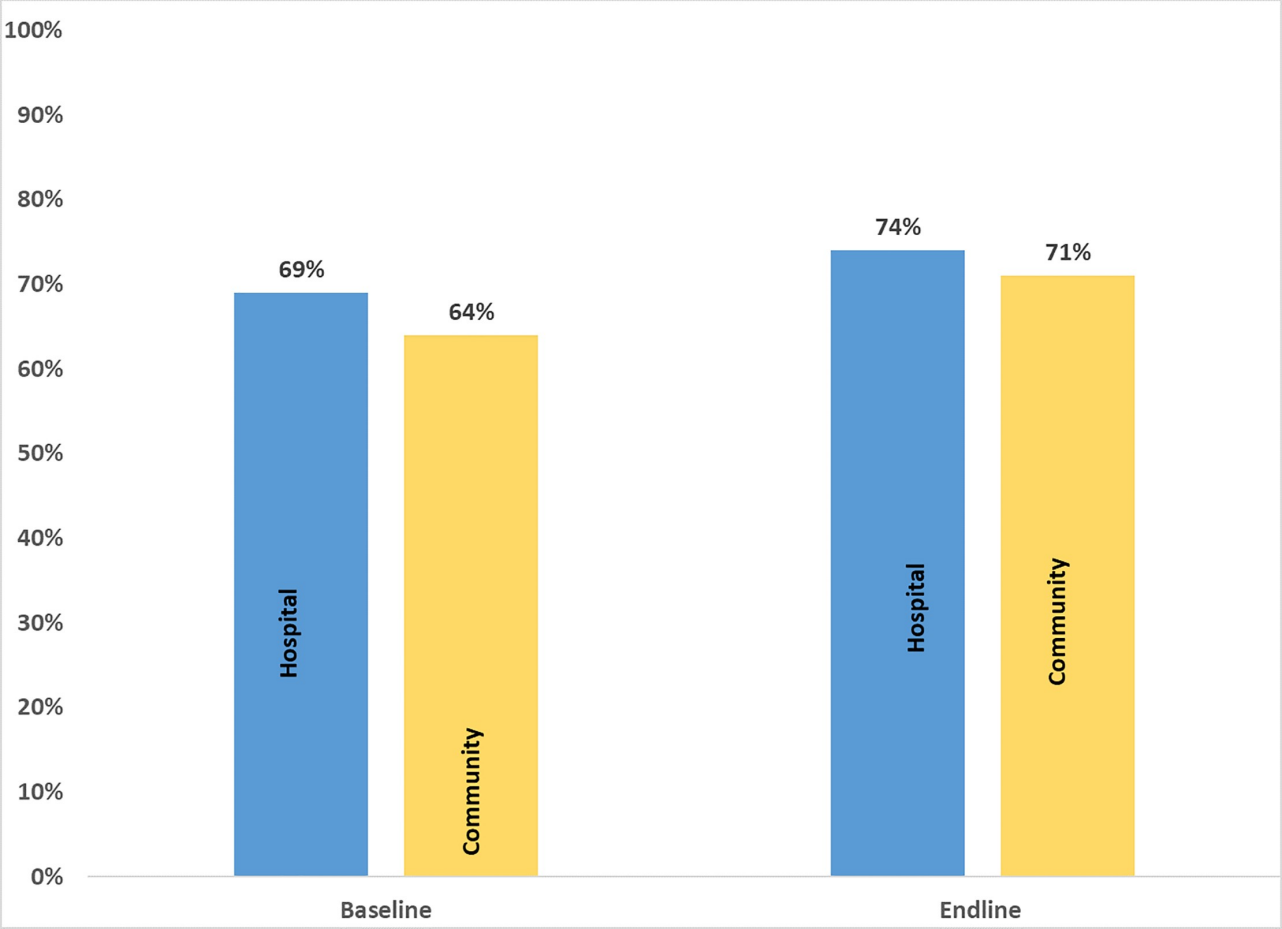
Compliance with IFAS among pregnant women. This refers to the level of maternal compliance with IFAS at both baseline and endline for both the control (hospital) and intervention (community) groups.

In this study, respondents who took at least 70% (5 tablets) of the expected dose of IFAS tablets in the week preceding the interview were considered compliant with IFAS as earlier explained. Fig 2 shows the levels of compliance with IFAS between baseline and endline across the two groups. There was an improvement in compliance with IFAS in both groups at endline. Levels of compliance increased by 8 percentage points (from 63.8% to 71.4%) and 6 percentage points (from 68.5% to 74.3%) in the intervention and control group, respectively. The intervention had a net effect of 2 percentage points (8–6) increase in compliance. However, it did not yield statistical difference since the DID between the two groups was 0.02 and the CI was -0.20, 0.24.

#### 3.2.2 Factors associated with maternal compliance with IFAS

Table 3 shows that the logistic regression to assess the effect of the intervention and other potential factors on maternal compliance with IFAS did not show statistical significance at p-value of 0.05 in compliance with IFAS between the intervention and control groups.

**Table 3:** Factors associated with maternal compliance with IFAS.

Variable	Odds Ratio	P-value	95% Confidence Interval	
**Community vs. hospital**	0.936	0.873	0.416	2.106
**Endline vs. Baseline**	1.634	0.172	0.807	3.310
Interaction (group, time)**	0.764	0.635	0.251	2.321
**Age group**				
20–29 years vs. <20 years	1.121	0.796	0.471	2.665
≥ 30 years vs. <20 years	1.133	0.833	0.354	3.631
**Highest education level**				
Secondary vs. Primary	0.900	0.736	0.489	1.658
Tertiary vs. Primary	0.680	0.550	0.191	2.413
**Occupation**				
Casual vs. Unemployed	1.289	0.526	0.588	2.827
Employed vs. Unemployed	0.759	0.402	0.399	1.445
**Single vs. Married**	1.528	0.388	0.584	4.000
**Income in USD**				
≥100 vs. <100	1.524	0.412	0.557	4.164
**Previous pregnancies**				
1 vs. 0	1.424	0.597	0.384	5.280
2 vs. 0	2.140	0.289	0.525	8.729
3 vs. 0	3.320	0.141	0.671	16.424
Multigravida vs. Primigravida	0.371	0.123	0.105	1.310
Catholic vs. Protestant	1.627	0.267	0.689	3.840

**DID

### 3.3 Side effects experienced with IFAS and their mitigation by respondents

**Fig 3:**
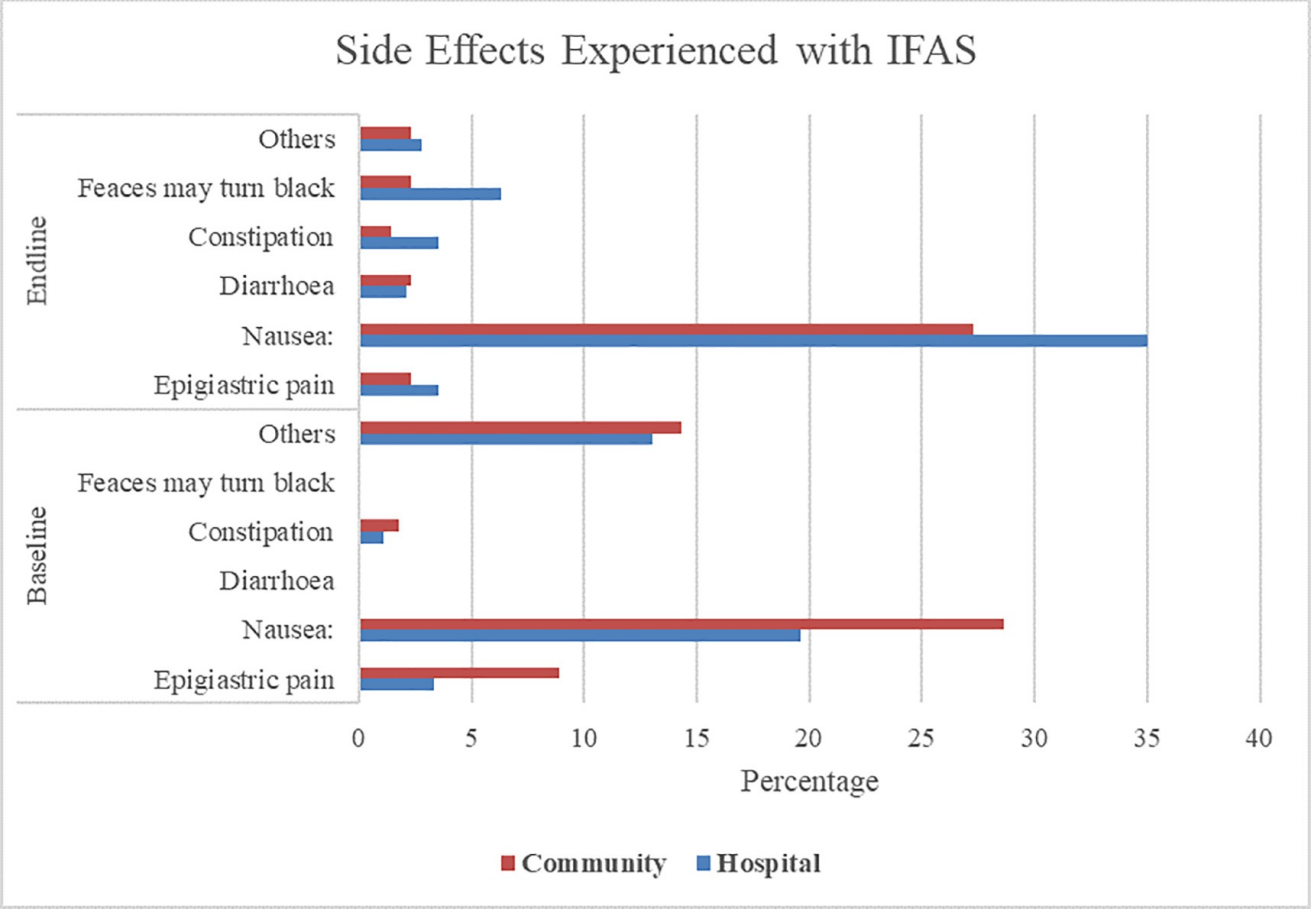
Side effects experienced by pregnant women with IFAS. This refers to the side effects experienced by pregnant women taking IFAS at both baseline and endline for both the control (hospital) and intervention (community) groups.

**Fig 4:**
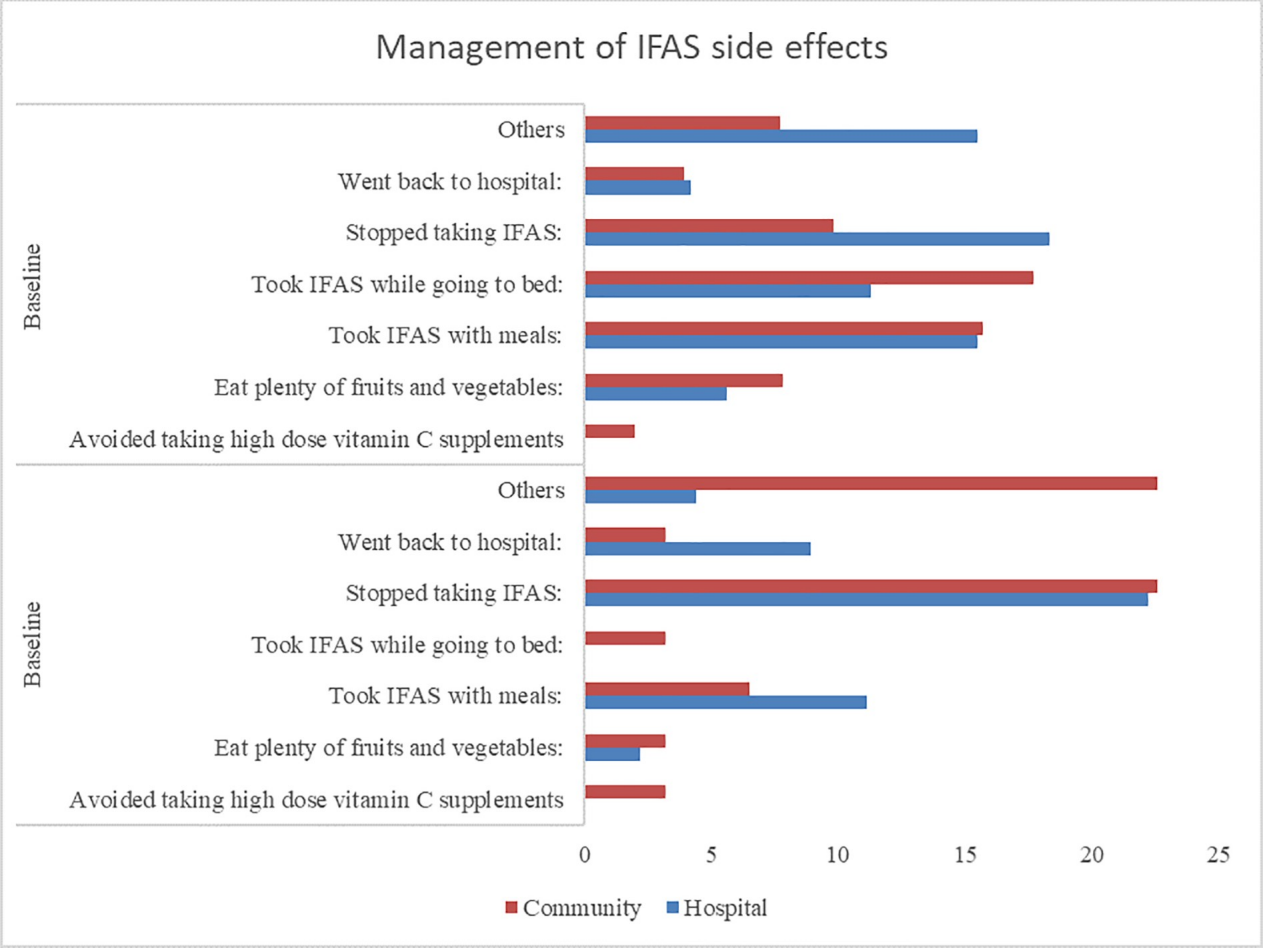
Mitigation measures of IFAS side effects by pregnant women. This refers to the measures that pregnant women taking IFAS used to mitigate IFAS side effects whenever they experienced them, at both baseline and endline for both the control (hospital) and intervention (community) groups.

The results in Fig 3 shows a comparison between baseline and endline proportions of side-effects experienced by each study group. There was increased awareness of the specific side effects of IFAS in both groups during the study period and respondents were better able to identify the side-effects associated with IFAS at endline than at baseline. For example, at baseline, none associated faeces turning black with IFAS, which is normal as a result of iron absorption and harmless. But at endline, they were able to identify it. In addition, the number of side effects reported was generally much lower in the intervention group compared to the control group at endline, unlike at baseline where the intervention group reported more side effects. Similarly, there was greater awareness on the mitigation of IFAS side effects in the intervention group than in the control group, as shown in Fig 4. Notably, in the intervention group, there was a greater decrease in the proportion of respondents who stopped taking IFAS (22.6%-9.8%) on experiencing side-effects. Moreover, there was greater change in the practices of managing IFAS side effects such as taking IFAS with meals (6.5%-15.7%) and taking IFAS at bedtime (3.2%-17.7%), in the intervention group compared to the control group.

## 4. Discussion

The aim of this study was to determine the effect of a community based IFAS distribution strategy on compliance with IFAS and identify side-effects experienced before and after study as well as the measures pregnant women used to mitigate these side-effects. The findings indicate that (1) there was a better improvement in compliance in the intervention group (2) there was increased awareness on IFAS side effects across the study groups (3) the intervention group reported fewer side-effects than the control group at endline (4) the intervention group reported better mitigation of side-effects than the control group at endline. These findings are consistent with literature on strengths of community based distribution of IFAS as a valuable platform in implementation of IFAS programmes [7].

Compliance with IFAS improved during the study period, indicating increase in IFAS uptake, with the intervention group showing higher improvement than the control group. This means there was an improvement in the proportion of women who consistently took the IFAS tablets as recommended. These findings were similar to a coverage of 67% reported in urban Nairobi County [40] in Kenya and 64.7% reported in India [41] respectively. However, the findings were higher than reports on IFAS coverage from low and medium income countries (LMIC) with similar rural settings namely, South Ethiopia (39.2%), Pakistan (38.3%), [38], Nigeria (37.5%) [42] and Western Ethiopia (20.4%) [20]. Nevertheless, lower coverage (18%) has been reported in a neighbouring County of Machakos, Kenya [43]. Elsewhere, Bilimale and colleagues suggested adoption of “more open, cooperative health professional-patient relationships” as critical to improving compliance [3]. Addressing all clients’ needs and issues through open communication is highly recommended. This has been achieved in this study by using CHVs during weekly IFAS distribution and follow ups of pregnant women at home. Since both HCPs and CHVs were trained in this study to provide IFAS tablets and counsel, both intervention and control groups yielded similar improvement in compliance levels although it was slightly higher in the intervention group. This highlights the importance of community-based distribution of IFAS and the need to adopt the community-based approach.

This study showed increased awareness on IFAS side effects in both groups. Studies have shown that accompanying health education with clear instructions on intake of IFAS substantially improves compliance [38, 44]. This is demonstrated in another Kenyan study where advice by health workers was independently associated with higher compliance [37, 45]. This implies that counselling on IFAS contributed to improving compliance among pregnant women in this study. Quality counselling should therefore accompany distribution of supplements.

Findings from this study showed a decrease in side effects reported at endline compared to baseline, especially in the intervention group. In addition, the study showed more awareness of mitigation of IFAS side effects in the intervention group. Studies have associated awareness of side effects of IFAS and their management with higher compliance [3, 20–22]. This is because side effects experienced on taking IFAS are associated with poor compliance. Many HCPs fail to inform clients about side-effects of IFAS resulting in either poor or non-compliance when they experience any slight discomfort [5]. Educating pregnant women on how to mitigate the side effects of IFAS often leads to higher compliance [3, 21, 46]. Many pregnant women stop taking IFAS tablets when they experience side effects. It is therefore critical to make pregnant women aware of the possible side effects and how to mitigate them in order to improve adherence to IFAS as indicated in other studies [3, 21].

This study showed better mitigation of IFAS side-effects in the intervention group. Although health workers may generally assume that talking about IFAS side-effects will make pregnant women not take IFAS, proper counselling and open communication leads to greater utilization as this study indicates. This was also demonstrated in a study among Indonesian women, who were not deterred by IFAS side-effects following prior discussion of likely side effects. This study indicates that side-effects and their mitigation measures is one of the IFAS topical areas that requires strengthening for quality IFAS counselling. This will eventually enable pregnant women to mitigate IFAS side-effects more effectively thus reducing their occurrence and improving their management whenever they occur, rather than discontinuing IFAS tablets which leads to poor or non-compliance.

This study had several limitations. (1) Following clients for a long period of time meant that some pregnant women were lost to follow up and this affected the power of the study. This was minimized by increasing the sample size by an estimated rate of loss to follow-up of 30%. Despite this consideration, there was a high loss to follow-up from 340 at baseline to 189 at endline mainly because of the extended industrial actions (strikes) among health workers in public health facilities during the study period. The industrial actions led to disruption of services in public health facilities that led to many clients seeking for antenatal and other health services from private and/or mission health facilities. (2) The study design did not involve randomization. The intervention was community based, using the natural setting and did not modify the environment of the respondents. The effect of this limitation was minimized by use of a control group to avoid extraneous variation resulting both from passage of time and from non-comparability of the test and control areas. Also, due to the low turn-out of antenatal clients in the Sub-County, consecutive sampling was used to include all accessible pregnant women as part of the sample. (3) Training of both HCPs and CHVs, instead of CHVs only, was also a limitation since it introduced confounding of the effect of the intervention. This was necessary as the CHVs needed to refer clients for other antenatal services and were being supervised and supplied with IFAS tablets by the nurses, so the nurses had to be trained as well. (4) The study results were prone to recall bias and subjectivity because the study greatly relied on verbal reports from the interviewees. This challenge was mitigated by training the interviewers as well as double questioning to identify any inconsistencies in the interview reports. (5) Generalizations of the study findings to other areas with different socio-demographic characteristics may be difficult since the study was restricted to one Sub-County.

## 5. Conclusion

Implementation of a community-based approach of IFAS distribution improved the maternal compliance with IFAS. Fewer side-effects were reported and were better mitigated especially in the intervention group. Use of CHVs for IFAS distribution is a potential approach for diversification of IFAS implementation in Kenya. It can be used to increase both demand and delivery of IFAS to pregnant women and to improve the low compliance levels. We recommend adoption of the use of CHVs to distribute IFAS supplements and follow up pregnant women to ensure improved IFAS uptake and compliance. This is a cost-effective strategy because CHVs are already operational in communities, so pregnant women will trust them. Findings from this study indicate and recommend that the time for integration of CBA into existing vertical health facility approach, to complement antenatal IFAS distribution, is now. This will further increase supplementation coverage and consequently reduce deficiencies of these crucial micronutrients among women and children, contributing to universal health coverage and achievement of sustainable development goal 3.
